# Review on the Relationship between Nano Modifications of Geopolymer Concrete and Their Structural Characteristics

**DOI:** 10.3390/polym14071421

**Published:** 2022-03-30

**Authors:** Fatheali A. Shilar, Sharanabasava V. Ganachari, Veerabhadragouda B. Patil, T. M. Yunus Khan, Naif Mana Almakayeel, Saleh Alghamdi

**Affiliations:** 1 Department of Civil Engineering, Jain College of Engineering, Belagavi 590014, India; shilarone@gmail.com; 2 Department of Chemistry, School of Advanced Sciences, KLE Technological University, Hubballi 580031, India; 3 Institute of Energetic Materials, Faculty of Chemical Technology, University of Pardubice, 53210 Pardubice, Czech Republic; iamveerabhadraa@gmail.com; 4 Research Center for Advanced Materials Science (RCAMS), King Khalid University, P.O. Box 9004, Abha 61413, Saudi Arabia; 5 Mechanical Engineering Department, College of Engineering, King Khalid University, P.O. Box 394, Abha 61421, Saudi Arabia; 6 Department of Industrial Engineering, College of Engineering, King Khalid University, Abha 62529, Saudi Arabia; halmakaeel@kku.edu.sa (N.M.A.); syalghamdi@kku.edu.sa (S.A.)

**Keywords:** nanomaterials, geopolymer concrete, compressive strength, SEM, XRD

## Abstract

The main objective of this review is to study some important nanomaterials and their impact on the performance of geopolymer concrete. This paper is an investigation into trends and technology in the development of different nanomaterials to develop higher structural performance geopolymer concrete. The effect of the alkaline to binder and sodium silicate to sodium hydroxide ratio on the performances of geopolymer performances is studied. The relationship between setting time and slump is evaluated through the ternary plot, the variation in compressive strength values is evaluated using the kernel density plot, and the relationship between split tensile and flexural strength is investigated using the scattering interval plot. Regression analysis is carried out among water absorption and bulk-density result values obtained from previous literature. As the molarity and alkaline to binder (A/B) ratios increase, the strength development of geopolymer concrete increases up to a specific limit. The addition of a small quantity of nanomaterials, namely, nano silica, nano alumina, carbon nano tubes, and nano clay, led to the maximum strength development of geopolymer concrete. Incorporating these nanomaterials into the geopolymer significantly refines the structural stability, improving its durability. The various products in GP composites emerging from the incorporation of highly reactive SEM, XRD, and FTIR analysis of nanomaterials reveal that the presence of nanomaterials, which enhances the rate of polymerization, leads to better performance of the geopolymer.

## 1. Introduction

Concrete is the second most often used substance on the planet after water, and it necessitates vast volumes of Portland cement (OPC). The manufacturing of OPC not only requires a large quantity of natural resources, such as limestone and fossil fuels, but also emits around 0.8 tonnes of CO_2_ for every tonne of cement clinker produced [1]. The cement sector ranks second in terms of greenhouse gas emissions, particularly CO_2_. CO_2_ emissions are predicted to reach a peak of 100% by 2020, compared to the present levels of output. The annual global cement output will be around 4.38 billion tonnes by 2050, with a 5% rise every year [2]. As a result, finding an alternate material to the current most costly, most resource, and energy demanding Portland cement is unavoidable. Geopolymers are gaining popularity as an alternative to Portland cement due to their reduced carbon footprint [3]. Geopolymers are formed by a polymerization process that involves a chemical reaction of alumina-silicate minerals in the presence of an alkaline media, resulting in the development of a three-dimensional polymeric chain. Geopolymers offer various benefits as binders, including strong mechanical strength, greater chemical resistance to corrosive environments, reduced creep and shrinkage, and resilience to high raised temperatures [4,5].

Nanotechnology is an innovative developing science in the area of civil engineering that is still in its early stages. Extensive attempts were made to integrate nanomaterials into traditional cementitious pastes to improve performance. Previous research has shown that incorporating nanomaterial into geopolymer concrete (GPC) enhances the geopolymerization and microstructure [6]. As a result of their exceptional and intelligent characteristics, cementitious materials mixed with nanomaterials led to high-performance structural components for various purposes in the building sector. In current years, multidisciplinary study emphasis has shifted to nanomaterials in construction applications [7]. The sol–gel technique to NS-solution may minimize the agglomeration of nanoparticles usually found in dry mixes, and the geopolymers (GP)s produced using this method demonstrated decreased micro-porosity and improved fire resistance performance [7,8,9]. The nanomaterials are extremely strong and have excellent physical and chemical characteristics. Several researchers have discovered several ways for producing nanomaterials. When nanomaterials are added to the polymer matrix, accessible alkaline solution is immobilized. Nanoparticles substantiate the gaps between binder grains, known as the filler effect [10]. The nanoparticles participate in pozzolanic processes, resulting in calcium silicate hydrates (C–S–H and N–A–S–H). Nanoparticles strengthen the connection among binder and aggregates at the interfacial transition zone [11]; ultimately, enhancing the bond strength properties of the mix. Nanoparticles enhance the flexural and tensile strength shear, providing crack arrest and a better interlocking bond among slip planes. The inclusion of ultrafine particles into Portland-cement paste and mortar produces properties that differ from ordinary materials [12]. The presence of nanoparticles in geopolymer concrete (GPC) leads to nanosized porosity at the interfacial transition zone (ITZ) among aggregate and cement matrices that significantly impacts performance. The nanosized particles significantly impact the macro and microstructure of GPC. Because of their high surface area to volume ratio, nanomaterials can operate as a pozzolanic and a nano-filler by filling the gaps among particles in C–S–H gel [13]. The large surface area of nanoparticles is critical for hydration. The nanomaterial improves early hydration and speeds up the creation of the hydration process [14,15]. Nanotechnology is an emerging discipline that aims to create new materials with improved characteristics and performance from a construction point of view. The main objective of this review is to study the different types of nanomaterials existing so far, and their performances in the strength development of geopolymer concrete (GPC). The synthesis of nanomaterials and their behavior to enhance compressive, tensile, and flexural strength is studied using different plots. In the following sections, attempts have been made to study the impact of nanomaterials on the matrix due to their partial replacement of industrial waste.

### 1.1. Nanofabrication Technique

Environmentally friendly, nontoxic, and safe chemicals are used in the “green synthesis” of nanoparticles. Nanoparticles made using biological or green technology have many properties, including high stability and enormous diameters [16]. Nanoparticles may be made using various methods, including chemical, physical, and biological methods. Chemical and physical techniques utilize many radiations, specific reductants, and potentially hazardous substances to the environment and human health. Nano concrete is defined as a nanomaterial or concrete containing nanomaterials with a particle size of less than 500 nm [15,16]. The addition of nanoparticles to concrete was thought to increase the strength of ordinary concrete. Nanoparticles improve the bulk characteristics of concrete, commonly known as the packing model structure. By refining the intersectional zone in cement and generating higher density concrete, ultrafine or nanoparticles may produce a fantastic filler effect. Their manipulation or change in the cement matrix system happens due to their function as an excellent filler, resulting in a new nanoscale structure [17,18,19]. The concrete microstructure removes micro-voids, porosity, and degradation due to the alkali–silica reaction. Nanomaterials then begin to emerge as a new binding agent smaller than cement particles. This enhances the hydration gel structure, resulting in a clean and stable hydration structure [20].

In addition, a novel concrete called nano concrete has been created using a mix of filler and a different chemical reaction in the hydration system. Nano concrete is durable and has improved performance [21]. Nanotechnology has been implemented in concrete since the early millennium. The use of silica fumes in traditional mix formulas improves the durability and strength of the material. Since then, nanotechnology has been developing to create a viable alternative to silica fume [22].

A common nano substance that replicates the effect of silica fume has been created using the nanomanufacturing idea. Nano silica is a relatively recent nanotechnology that has been utilized as a replacement for silica fume [23]. Many nano-based particles have been created for use in concrete since the discovery of nano silica. Nanomaterials utilized in nano concrete include nano alumina, titanium oxide, carbon nanotubes, and polycarboxylates. To detail an understanding of the effect of nanomaterials, one needs to study the synthesis of nanomaterials, their structural performances such as setting time, flow, compressive, tensile, flexural strength, water absorption, and bulk density and application of nanomaterials in practice.

### 1.2. Production of Nanomaterials

Since the advent of nanotechnology in the late 1960s, the notion and concept of manufacturing nanomaterials have evolved. Compared to micro-based materials, the nano size of nanoparticles has a more substantial influence on filler [24]. It is considered successful when nanoparticle production impacts parent material purity or basic chemical composition. The first is a top-to-bottom method, and the second is a bottom-to-top approach. The two techniques were chosen based on their applicability, affordability, and knowledge of nano behavior [10,25]. Milling is a method used in the top-to-down approach. The milling approach was chosen due to its availability and feasibility since any change may be made directly without the need for any chemical or electrical equipment. The top-down technique states that enormous structures can be reduced in size to nanoscale while retaining their original characteristics or chemical composition with no change in atomic-level control [23,26]. In other words, mechanical attrition and etching methods break down bulk materials into nanoparticles. This approach is often used in large enterprises. Nanoparticles are generated in large quantities using the milling process because it is cost-effective and straightforward to maintain due to more mechanical instruments and more negligible chemical modification [27]. Another phrase for the top-down technique is the modern method in nanomanufacturing. However, with a top-to-bottom method, the consistency and quality of the end output are uneven.

Although there are drawbacks to the top-down method, the quality of nanoparticles may be enhanced by modifying milling procedures such as the number of balls used, the kind of balls used, the speed of milling, and the type of jar used. High energy ball millings are frequently used to produce nanomaterials, nano grains, nano alloys, nanocomposites, and nano quasi-crystalline materials [28,29]. John Benjamin was the inventor of the milling process for generating oxide particles in nickel superalloys (1970). His first milling effort was when he changed and strengthened an alloy component for high-temperature construction. Plastic deformation, cold welding, and fracture are variables that influence the deformation and transformation of materials during milling [29].

Milling is the process of mixing numerous particles or materials and converting them into new phases of material composition, in addition to breaking them into smaller bits. The final output of the milling process is usually flaked in shape. However, refining can be performed based on the ball selection and milling type. However, most nanomaterials utilized in concrete, such as nano silica, nano alumina, and nano clay, are generated from the bottom up [2,30]. A bottom-to-top method is used when materials are created from atoms or molecular components by assembly or self-assembly. It is also referred to as molecular nanotechnology or the molecular manufacturing process, and it has more indirect uses such as synthesis and chemical formulation. The size and form of nanoparticles generated by a bottom–up method may be defined and controlled using a chemical synthesis process. The difference between this top-down strategy is that the bottom–up approach produces more homogeneous and tidy nanoparticle structures. In other words, because the atoms or molecules are precisely organized or crystalline, bottom to up also generates new nanocrystals [31,32]. Electronic conductivity, optical absorption, and chemical reactivity are the approaches used. The bottom to up method allows for size reduction and tidy surface atom creation, which results in a significant shift in surface energies and morphologies. Typically, using this approach, nanomaterials may be broadly adapted in the circumstances such as enhancing catalytic capability, detecting wave ability, and new pigments and paint with self-healing and cleaning characteristics, and so on. However, the drawback of the bottom to up method is its high operational expense, the need for knowledge in chemical applications, and its restricted applicability since it is intended primarily for laboratory use [8,24,33]. However, nanoparticles produced using this process are ideal for advanced applications such as electrical components and biology. Finally, in addition to assessing the impacts of nanomaterials, distinct techniques to produce nanomaterials for use in ultra-high-performance concrete were described [17,33].

#### Sol–Gel Technique

**Figure 1:**
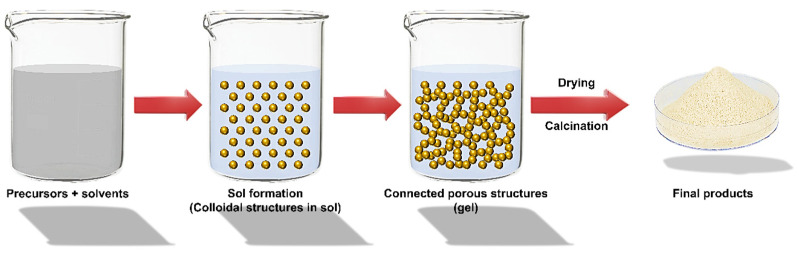
Scheme of Sol–gel synthesis.

Creating nano metal and metal oxide (MO) materials necessitates a high level of synthetic inventiveness. Although a balanced synthesis technique can be developed, there is a constant element of serendipity in nanomaterials. In the last few decades, a wide range of nanomaterials has been created using this traditional method [1,34]. The ceramic technique entails combining and crushing component granules, oxidizing, carbonating, and heating other chemicals at high temperatures. When necessary, transitional grinding is used. Precipitation and precursor methods, ion exchange and sol–gel techniques (Figure 1), topochemical methods, and others are all effective chemical methods for synthesizing oxides.

The relevance of the sol–gel method and chemistry in materials production has been steadily increasing [35]. The chemical reactions of volatile metal precursors, generally alkoxides in conjunction with alcoholic solution, sequent inside the conforming hydroxide, are commonly employed to create metal oxide. Hydroxide molecules condense and are linked to eliminating water, which helps organize a robust base network. Gelation occurs when hydroxide molecules create a network-like structure, resulting in a thick porous gel. A chemical compound shows the gel with a three-dimensional skeleton close to the reticular pore. The evaporation of solvents during the drying of the gel results in the creation of ultrafine metal OH powder, which contributes to the conforming ultrafine powder of the MO [36,37].

Because this technique starts with a nano-sized material and reacts on a nanoscale scale, nanometer material creation is a virtual certainty. Because of the existence of metal-oxygen linkages matching to alkoxide precursors, sol–gel methods have proved appropriate for generating only MO, and the required gels are essentially metal hydroxides or oxides [38]. A sol–gel processing approach was created in a recent study to generate a vast range of ceramic materials, including Al_2_O_3_, Fe_2_O_3_, SiO_2_, TiO_2_, and others. However, several investigations have demonstrated that non-oxide powders may be made from organometallic precursors other than alkoxides via sol–gel processes. For manufacturing nano-powders of MO ceramics, sol–gel techniques provide several advantages over other approaches [39,40,41]. As a result, the invention’s repeatability results from the stable molecular amalgamation of the starting components. The sol–gel method also has a strong potential for generating employment in sectors at more excellent rates. The scaling up of several industrially relevant MO nanoparticles has been successfully developed [42,43].

### 1.3. Structure Interaction of Nanomaterials in GPC

Nanomaterials such as nano-SiO_2_, CNTs, and nano-TiO_2_ can positively impact the polymerization processes and the physical structure of N–A–S–H in GPC. Nano-SiO_2_ can modify the shape of GPC by creating more N–A–S–H gel and fewer ettringite crystals, in addition to being a dense substance [10,25,40,44]. Surface energy, morphology, and chemical reactions in GPC can all be affected by the dramatic increase in surface area of nano-SiO_2_. Nano-SiO_2_ helps form tiny-size crystals and clusters of N–A–S–H during the pozzolanic reaction due to its small particle size and high surface fineness [45]. The relatively small particle size of nanoparticles compared to traditional concrete cementing ingredients may allow for more excellent void filling and other beneficial filler effects; the filler effects generate a geopolymer microstructure with enhanced density and reduced density porosity [46,47].

**Figure 2:**
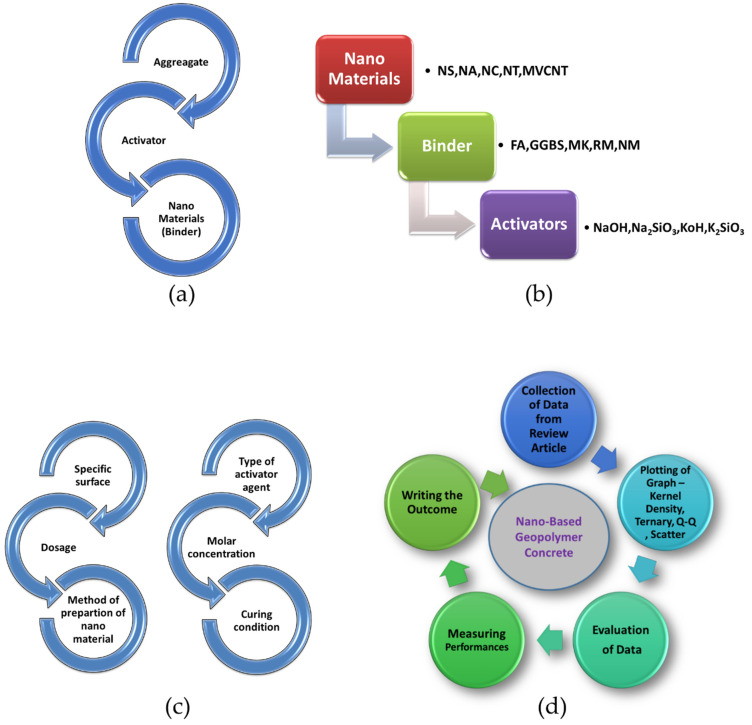
Schematic representations of; (**a**,**b**) The different ingredients of GPC and nanomaterials used in GPC; and Schematic representation of usage of nanomaterials. NS—Nano Silica, NA—Nano Alumina, NC—Nano Clay, NT—Nano Tube, MVCNT—Multi-Walled Carbon Nano tube. FA—Fly Ash, GGBS—Ground Granulated Blast-furnace Slag, MK—Metakaolin, RM—Red Mud, NM—Nano Materials. NaOH—Sodium Hydroxide, Na_2_SiO_3_—Sodium Silicate, KOH—Potassium Hydroxide, K_2_SiO_3_—Potassium Silicate, and (**c**) various factor that influences the performances of GPC. (**d**) Methodology adopted for conducted research review.

Nanoparticles arrange themselves in an efficient close-packed form. A dense collection of congruent spheres in an endless and regular configuration is known as close-packing comparable spheres in geometry. Nanomaterials can function as fillers to create a dense and less permeable mortar microstructure; they can also operate as nuclei to aid the development of polymerization products and, therefore, encourage the construction of high-density sodium alumina silicates hydrate (N–A–S–H) structures, according to the researchers [15,31,48]. Nano-SiO_2_ in concrete can make the microstructure more homogeneous and compact than regular cement. Nano-SiO_2_ improves concrete microstructure in four ways: (a) as a nucleus, (b) by producing improved calcium silicate hydrate (C–S–H), (c) via regulated crystallization, and (d) by filling micro-voids. When nano-TiO_2_ is combined with cement, the porosity of the concrete is reduced as well. Nano-TiO_2_ can change the pore size distribution and reduce overall pore volume by progressively filling up the pore space surrounding them as hydration continues [6,49]. Concrete having nano-TiO_2_ has a finer pore structure than concrete with nano-SiO_2_. As a result, geopolymer concrete having nano-TiO_2_ may be more resistant to the entry of harmful chemicals than GPC containing nano-SiO_2_. Figure 2 shows Figure 2a the different ingredients of GPC; Figure 2b the nanomaterials used in GPC; Figure 2c various factors that influence the performances of GPC; and Figure 2d the methodology adopted for the conducted research review.

## 2. Types of Nanomaterials Used in GPC

**Figure 3:**
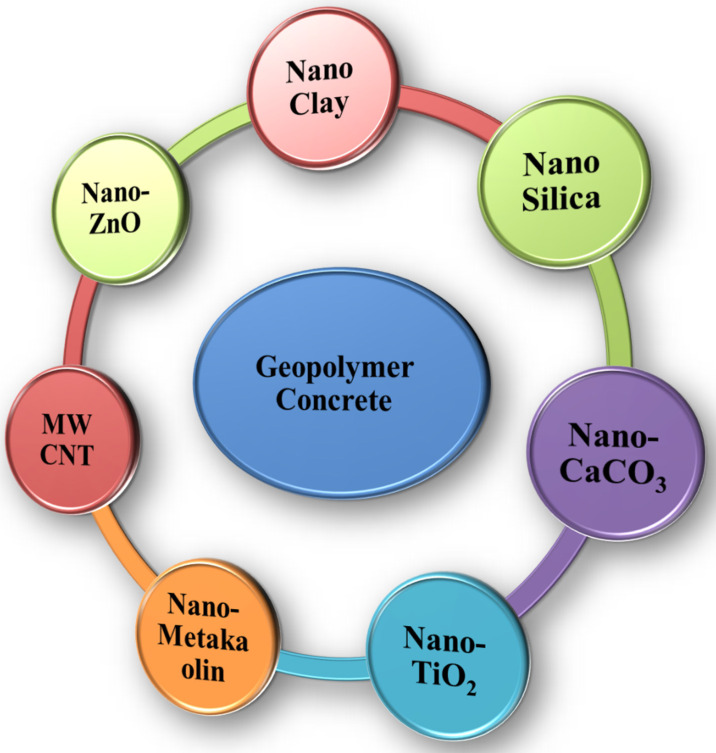
Schematic representation of different nanomaterials used in geopolymer concrete.

Figure 3 shows the different nanomaterials used in GPC. Nanomaterials are gaining popularity in science and engineering domains. The inclusion of nanomaterials in GPC mortars might improve the overall structural performance of GPC. Nanomaterials are divided into two categories: pozzolanic-based nanomaterials such as nano silica, nano clay, and nano alumina; and secondly, fiber-based nanomaterials such as carbon nanotubes (CNT) and carbon nanofiber. Compared to ordinary concrete, adding nanoparticles to geopolymer-based mortar results in more significant strength development [11,50].

Through their morphologies, the fiber-like nanomaterials feature needle action systems. The needle shape promotes the reduction in the growth of cracks and prevents their spread by improving the tensile strength. The pozzolanic reactivity of nano clay was strong, and it had a substantial impact on mechanical performance [6,51].

### 2.1. Nanomaterials Used as Binders in GPC

#### 2.1.1. Nano Silica (NS)

The degree of fineness is 60–65 m^2^/g. The GP with 2.5% NS addition had the best strength even at increased temperatures, with no visible cracks and 50% mechanical strength. Due to high specific surfaces and better particle size, the matrix flowability is reduced with less setting time. The combination filling effect of NS from better particle packing and the different reaction products resulted in a denser binding matrix, decreasing porosity, and enhancing CS. A GP with an optimum solid-to-liquid ratio and incorporated NS has more structural water and gels, which aids structural growth. The findings also demonstrate that nanotechnology composites have many possible uses. With NS or SF, the matrix increases the silica species, resulting in more silicate oligomers. The transformation of silicate oligomers to a polymer network takes time [24,34,45,49,52].

As a result, the paste setting process is slowed. It was discovered that NS does not dissolve fully in hydroxide solution, resulting in decreased viscosity and improved workability. Slump value increases significantly with increasing NS concentration in FA-based geopolymer mortar. A decrease in slump value of 18% to 46% with an increase in NS ranging from 1% to 3%. The fineness of NS, which leads to higher water requirement, was cited as one of the demerits for the slump value decrease [33,52].

#### 2.1.2. Nano-TiO_2_ (NT)

NT can accelerate the early-age hydration of Portland cement, improve compressive strength (CS) and flexural strength (FS), and increase abrasion resistance in concrete. The inclusion of NT increases GP production, resulting in a denser microstructure with fewer fractures. It increases GPC carbonation resistance and decreases drying shrinkage. The inclusion of NT with a smaller particle size increased the CS of the mortars at all ages [48,49].

#### 2.1.3. Nano Metakaolin (NM)

NM plays a vital role in the strength development of GPC as binder. According to past studies carried out by various researchers, they concluded that CS at 3 days in ambient conditions was around 70–80% of 28 days strength for the same specimens when NM was used to prepare GPC as the binder. With the addition of NM, the GP mortar Si/Al ratio falls while the CS increases. As a result, the higher the Si/Al ratio, the lower the resilience of GPC [53,54,55].

#### 2.1.4. Carbon Nanotubes (CNT)

Carbon nanotubes (CNT) are hexagonal sheets of carbon atoms that have been formed into a cylinder. The addition of MVCNT to GGPC improves the tensile characteristics considerably. Because of the large specific surface area of CNT particles and the strong van der Waals forces exposed to the aspect ratio; they are prone to agglomeration. The aspect ratio of CNT varies from 1000 to more than 2,500,000. The oxidation detritus on the CNT surface may be removed using NaOH solution, which improves dispersion. As a result, the alkaline solution used in GP formation can behave as a surfactant, enhancing deagglomeration. The addition of even a tiny quantity of CNT improves the mechanical properties significantly. CNT has macro and micro impacts, such as bridging influence, which helps inhibit crack propagation and achieve load transfer. Enhancing concrete characteristics and bond strength using a mix of CNT and nanoparticles is typically adequate [50,54,55].

#### 2.1.5. Multi-Walled Carbon Nanotubes (MVCNT)

When MVCNT is added to GGPC, it enhances nucleation sites, and accumulation of MVCNT results in C–S–H gel, which results in high hardness, better pore topologies, control of nanoscale fractures, and lower drying shrinkage of GGPC. Furthermore, the well-dispersed and homogeneous distribution of CNT substantially enhances the particle packing of GGPC, resulting in their exceedingly compact development [5,7,34]. These activities halt and bridge fracture development, and inhibit crack spread. This, in effect, leads to an increase in chloride penetration resistance. The introduction of CNT has resulted in a considerable increase in the mechanical characteristics of GPC. Furthermore, the homogenizer enhanced the capacity of CNT particles to function as filler material and strengthened the paste and enhanced FS [50,56,57].

#### 2.1.6. Nano Clay (NC)

Nanomaterials improve the CS of hybrid FA/slag in the GP matrices by enhancing the density and hydration of the polymerization process. GPC is impacted by the binder chemical composition and fineness of binder. Silica and alumina content in the binder to a specific limit (up to an optimum limit) leads to leaching in an alkaline environment, leading to a higher CS. It is more logical to link the CS findings to the gel/space % of ratio. NC produces a denser mixture with a more reduced porosity and water content than the control mix. Researchers have extensively studied the use of NC particles in binder grains, promoting the pozzolanic reaction and enhancing mechanical performance by decreasing the size and porosity of the cement matrix due to its high surface area to volume ratio and capacity to display remarkable chemical reactivity. NC addition to GP paste can enhance polymerization. The optimal nano clay content results in increased polymerization, whereas more NC concentration results in an inactive state with no further improvement. Authors reported enhanced polymerization formation, which was ascribed to the production of micro-dispersion by NC in the GPC, in research exploring the influence of nanomaterials on freeze and thaw resilience of slag-based GPC [5,46,53,58].

#### 2.1.7. Nano-CaCO_3_ (NCC)

During the polymerization process, the addition of NCC to GP paste acts as a potential catalyst. It helps to speed up geopolymerization by adding NCC, forming a new calcium silicate hydrate (C–S–H). Also, with the addition of this nanomaterial to GP and it enhances the chemical reaction and a continuous geopolymeric reaction finally results in a denser microstructure [59].

#### 2.1.8. Nano-ZnO (NZ)

The addition of NZ enhanced the homogeneity of the GP matrix; the density and compactness of the network because of the improved interfacial adhesion of the GPC and NZ filler. It is suitable to add a small quantity of NZ (0.5%) to the GP used in building since the resultant composites have a CS virtually equivalent to the CS of ordinary concrete (40 MPa) [39,49,55].

### 2.2. Geopolymerization Process

Geopolymers are related to inorganic polymers. The chemical makeup of these materials is nearly identical to that of zeolitic materials, with the sole difference being that the microstructure is amorphous rather than crystalline. The polymerization process involves a quick reaction rate in activator chemicals on Si–Al ions, forming a 3-D polymeric chain [60]. The polymerization process involves a quick reaction of between activator agents on Si–Al minerals, which leads to the formation of a 3-D chain and the Si–O–Al–O link. The basic principle of the polymer is that when Si/Al-rich materials are combined with an activator chemical, a Si–O–Al–O link is formed by polymerization [61]. The polymerization process comprises of a significantly quicker chemical reaction in the presence of an activator on Si–Al-rich materials, leading to the formation of a 3-D polymeric chain and a ring structure involving the creation of a Si–O–Al–O framework. The development of GGPC as a poly-condensation from Si and Al, along with a high alkali content, resulted in strength development [40,62]. GPC is amorphous, like synthetic zeolites, and has a chemical structure comparable to the zeolitic structure. Geopolymers are composed of a polymeric Si–O–Al framework, unlike zeolites, with alternating Si–Al, forming tetrahedral shapes joined together in 3-D by oxygen atoms. A decrease in the Si/Al ratio increases the surface area of the GGPC, which is advantageous for the adsorption phenomena [34]. The inclusion of a crystalline form of Al_2_O_3_ lowers the Si/Al ratio in the matrix, resulting in a greater degree of geopolymeric gel rearrangement, promoting polymerization. Due to the presence of an amorphous form as opposed to the nano-crystalline Al_2_O_3_ phase, it was thought that NS impacts the polymerization reaction, whereas nano-Al_2_O_3_ presumably does not contribute to this process and affects the function as nanofillers [63].

The addition of nano alumina to GGPC causes substantial changes in the kinetics of polymerization, which may be because nano alumina particles act as a catalyst in the formation of polysialate. Growth is achieved by allowing the nano alumina particles to interact more efficiently in the responding system many times [64]. This causes the nano alumina particles in the reacting system to interact more effectively, leading to the formation and development of N-A-S-H gel. GP based on FA and nano alumina determined a concentration of 2 wt.%. The % NS and nano alumina was optimal for speeding the geopolymeric process while filling gaps to generate denser matrices. They also demonstrated that nano alumina particles, in addition to functioning as a filler material, accelerate the geopolymeric process and improve the geopolymer microstructure. As a result, they appear to be highly successful in enhancing the nanocomposites interfacial bonding quality. As a result, nano alumina, a high-aluminum substance, should be used in conjunction with GPC made from low-aluminum precursors such Rice Husk Ash (RHA) to speed up the polymerization [18,65].

#### 2.2.1. Effect of Alkaline to Binder Ratio in Strength Development

A mixture of NaOH and Na_2_SiO_3_ is the most often utilized activator in the production of GGPC. Potassium silicate and potassium hydroxide must also be utilized. Activators have a significant influence on the polymerization process. Polymerization occurs rapidly when the alkaline activator dissolves the Si and Al from the binding material to create the matrix [66,67]. The reactivity of FA is enhanced by employing an activator made from NaOH and Na_2_SiO_3_. On the strength property, the GGPC performed better when a superplasticizer was used. When compared to 0.30, the alkaline to binder (A/B) ratio of 0.4 had the highest CS. The author researched GPC using FA as a binding material, varying the A/B ratio from 0.25 to 0.40. The addition of 5% NS to GGPC raises the SiO_2_/Na_2_O ratio ranging from 9.85 to 11.25, demonstrating the dissolution of the materials, followed by the alliance of the oligomer with chain formation of N–A–S–H. Furthermore, by adding 5% NS, the Si/Al ratio is increased from 2.2 to 2.49 [68].

The Si/Al ratio has a significant impact on the mechanical and structural characteristics of GGPC, with a high Si/Al ratio resulting in increased mechanical and chemical stability and increased Si–O–Si bonds and residual silica, which enhances Si–O–Si bonds and residual silica, at the interfacial transition zone. The NS primary purpose is to speed up geopolymerization [13,16,55,69]. The presence of nanomaterial leads to improvement in dissolving the matrix which gains a high-energy surface by increasing Al(OH)^−4^ and Si(OH)^−3^ ions, and further it increases an creation of number of unsatisfied Si–O and Si in the NS surface and, therefore, these unsatisfied Si–O and Si located in the surface of the NS, actively involvement in nucleation site of Na_2_O–SiO_2_–Al_2_O_3_–H_2_O gel [69,70].

#### 2.2.2. Influences of the Ratio of Sodium Silicate to Sodium Hydroxide (SS/SH)

For GPC, the SS/SH was considered 2 and 2.5. The highest CS value was recorded for two ratios, and a strength of 30 MPa was attained on day three. Researchers evaluated the use of FA as a binding material, varying the SS/SH ratio from 1.75 to 3. They discovered that the highest CS was obtained at 2.5 ratios at room temperature. Between 2.5 and 3, there was just a minor improvement in the CS value. The influence of temperature as a curing condition on the SS/SH ratio was examined for temperatures of 60, 75, and 90 °C for exposure times ranging from 24 to 48 h. They discovered that an SS/SH ratio of 2.5 at 75 degrees for 24 h resulted in the highest CS value [71,72,73]. When GP is subjected to ambient temperature and oven-cured conditions, the CS value increases with increasing SS/SH ratio up to an optimal point, after which the CS begins to decrease. The highest CS value for 14 M with the oven-cured specimen is 35.7 N/mm^2^ after 56 days. The highest CS value for 56 days in 16 M with an ambient cured specimen is 25.8 N/mm^2^. The highest CS value was discovered for specimens exposed to oven-cured temperatures rather than ambient cured temperatures. Table 1 shows the summary of the literature study carried out by a researcher on GPC with different nanomaterials as the binder [74].

## 3. Influences of Nanomaterials on Fresh and Hardened Properties of GPC

### 3.1. Relationship between Setting Time and Workability of Mortar Using Nanomaterials

**Figure 4:**
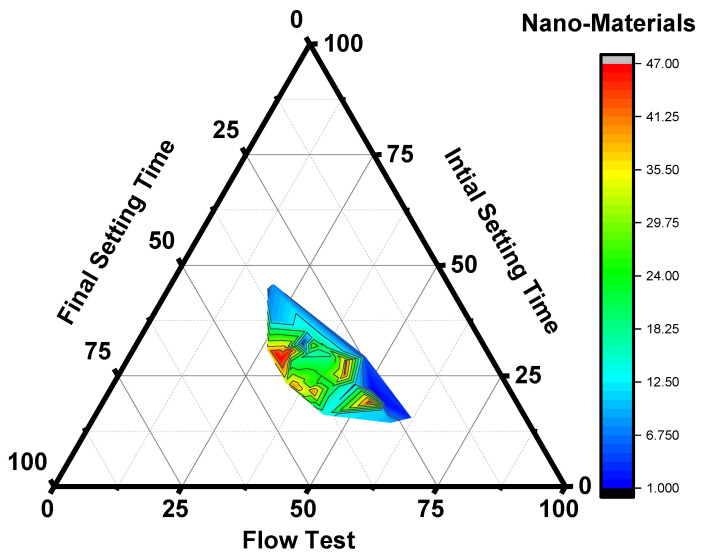
Ternary plot for flow test (mm) and setting time (min).

Figure 4 shows the ternary plot representing the relationship between flow test (mm) and initial and final setting time (min). The harsh and stiff matrix of GP dramatically depends on the types of activator agent, molar concentration, and alkaline to binder ratio (A/B). The fluidity of GP paste can be enhanced after the addition of an admixture. Flow test and setting time test value collected from past literature papers (Table 1) represented in the ternary plot; red color contour zone in the ternary plot indicates that the maximum results value of the flow test and setting time test (from past literature) lay in the red color area of contour zone. The density in the red zone ranges from 37.50 to 47.00. The maximum among other zones that indicate maximum results from the data is a cluster at 37.50 to 47.00 density. The green color contour zone indicates the second maximum cluster with density 12.50 to 37.50, followed by the blue color contour zone indicates the last cluster with density 1:00 to 12.50.

The higher the range of cluster density indicates that the maximum values of flow test and setting data lay in that zone. Most of the past research data carried out with nanomaterials as an additive in GP concrete had a density of 37.50 to 47.00 with their flow, and initial and final test values lay in the range from 50 to 80 values (obtained from the ternary plot). The presence of NS has a considerable impact on the setting time of GPC. Because of the high nanoparticle action of NS, the addition of NS resulted in a noteworthy reduction in the setting time of metakaolin-based GPC, which further expedited the polymerization process [27,45,46,47,48,49,50]. As the SiO_2_/Na_2_O ratio increased, the initial and ultimate setting durations of NS metakaolin-based GPC began to increase. It was discovered that a more extensive sodium silicate content lead to the paste matrix having longer setting times [75].

The use of retarders delays polymerization development without sacrificing the strength qualities. Setting times were decreased by adding NS and nano-Al_2_O_3_.The workability of GP products was reduced by using NT, regardless of the underlying material type. Adding 3% and 5% NT particles considerably decreased the workability of the GPC by 21.86% and 31.12%, respectively.

The inclusion of NT particles reduced GP setting time, which might be ascribed to pore-filling effects. Setting time of GPC with NA addition shows just a slight reduction. Because nanomaterials are included in geopolymers, higher pozzolanic is attributed to a quicker setting time of geopolymers [76]. This results in a more effective form of a rigid 3-D network of monomers, which reduces GP setting time. Some nanomaterials pozzolanic properties (e.g., NS, NA, and NC) may speed up the polymerization of the binder, reducing workability. While changing the w/b ratio may enhance workability, it harms hardened GPC mechanical characteristics and durability. Superplasticizers can alter the surface of both nanomaterials and FA particles, improving the workability of the resulting combination. However, strong alkali used to activate the FA particles may decrease the superplasticizers effectiveness [37,77].

### 3.2. Impact of Nanomaterials on Development of CS

**Figure 5:**
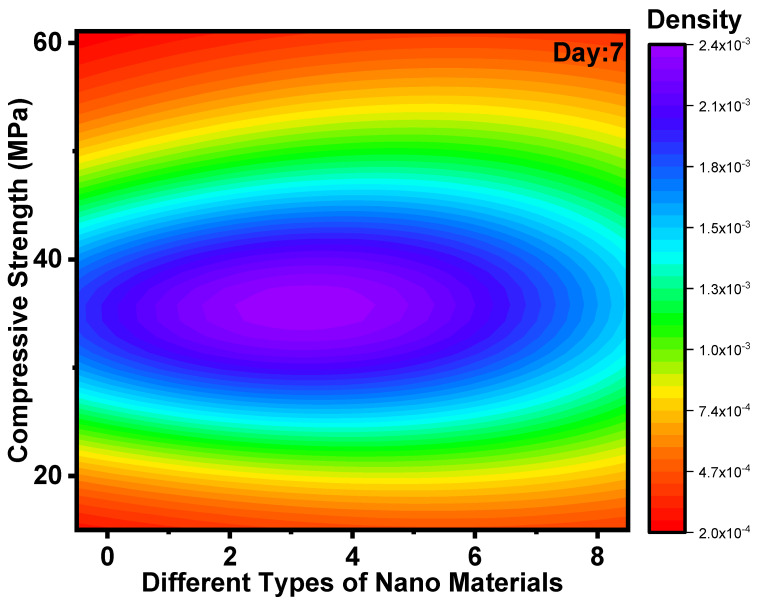
Kernel density plot for CS for day 7.

**Figure 6:**
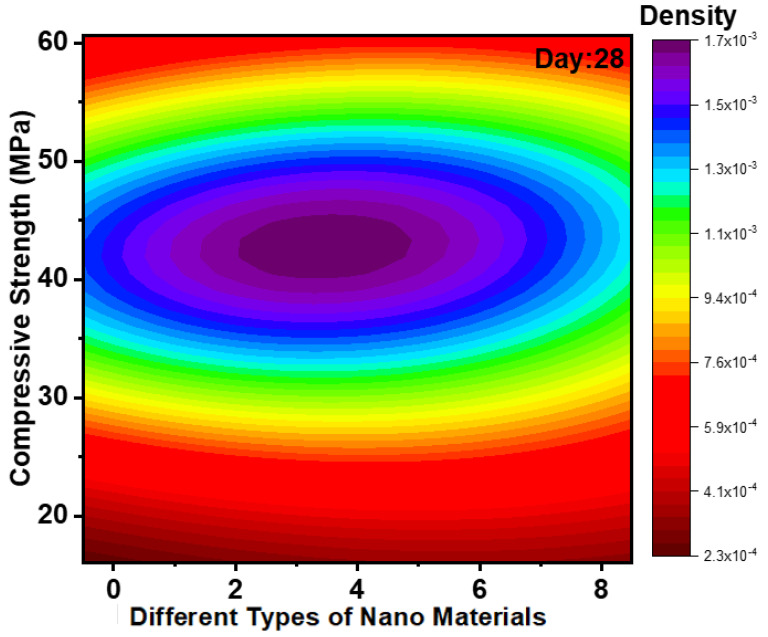
Kernel density plot for CS for day 28.

Figure 5 and Figure 6 show the Kernel density plot for CS for days 7 and 28. CS for different mixes of GPC for 7 days from kernel density plot observed that two contours are visible for the first contour maximum values ranging from 25 to 42 MPa. The data from about 40 research papers represented in kernel density plots showed that for 7 days, CS is in the range from 25 to 42 MPa. The CS and FS of GPC rise when NS or NA (i.e., NS as 1% and NA as 2%). These are due to an increase in the quantity of GP matrix products and improved pore architectures of the combination with NS or NA. As comprehensive information shows, several others have reported enhanced mechanical characteristics of various types of GP with NS or NA added. The effects of NC as a binder addition on the CS of GP are comparable to those shown in GP with NS and NA, where the CS of GPC rises with a specific dose of NC [38]. In recent years, the uses of carbon-based nanomaterials in GP have piqued the interest of researchers, as have graphene of various forms. CS for different mixes of GPC for 28 days from kernel density plots observed that two contours are visible for the first contour maximum values ranging from 35 to 50 MPa. The data from about 40 research papers represented in kernel density plots showed that for 28 days, CS was in the range from 35 to 50 MPa. Nanomaterials improve the CS of hybrid FA/slag GP concrete matrix by enhancing the polymerization response by growing the generated hydrated gel and density [78].

It was discovered that the CS of geopolymers is determined by the material type and fineness. Fine materials cause more Si and Al leaching in alkali environments, resulting in a more excellent composition. Compared to the control combination, nano clay generates a denser mixture with lower porosity and WA. The addition of 0.02% CNTs increased compressive strength to the highest levels among the mixes containing different CNTs individually. Compared to the control mix, the enhancement values at 28 and 60 days were 81% and 57%, respectively [21,68,79]. Carbon nanotubes entering the GP matrix increase matrix uniformity while increasing compressive strength. According to the literature, CNT function as bridges to prevent micro fractures from spreading. Addition of small amount of CNT makes it possible to improve the CS of FA-GPC. The tremendous increase in 28-day CS was produced by blending nano-SiO_2_ at 6 wt.% in a low-calcium FA-GP concrete specimen that included varied doses of colloid nano-SiO_2_ (up to 10%, by weight of the binder). The 28-day CS of an FA-GP paste incorporating 2 wt.% nano-Al_2_O_3_ increase from 24 to 30 MPa (by 25%) and observed more refined microspore structure, attributed to the nano filler effect (from 30.3 to 43.0 MPa) percent CNF, and attributed the increased strength to the crack-bridging action. The addition of 2 wt.% NC increased the 28-day CS of an FA-GP paste by 23.4% (37.2 to 45.9 MPa). Nano clay enhances the degree of polymerization, accountable for improved strength development (from 33.6 to 41.4 MPa at 28 days) [29,58,80].

### 3.3. Impact of Nanomaterials on STS and FS

**Figure 7:**
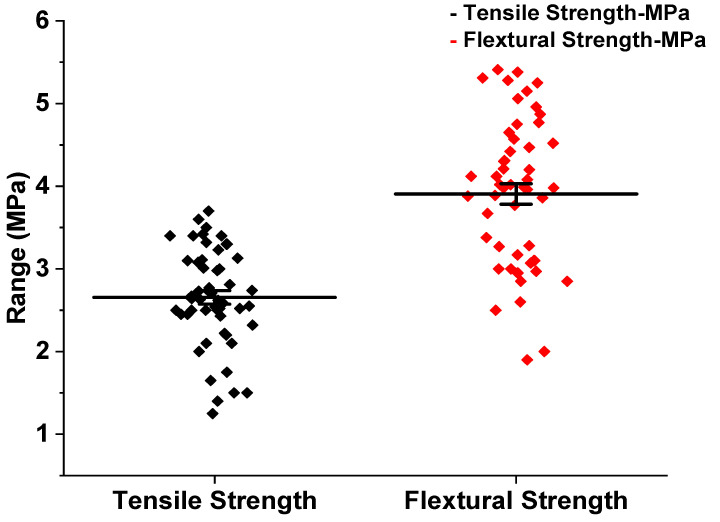
Scatter interval plot for STS (MPa) and FS (MPa).

**Figure 8:**
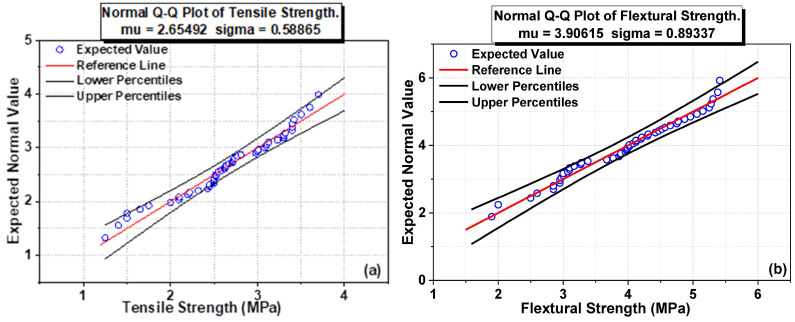
Normal Q-Q plot for ST (**a**) and FS (**b**).

Figure 7 and Figure 8a,b show the scatter interval plot for STS, FS, and Normal Q-Q plot for ST and FS. STS and FS values for 7 days of GPC collected from various literature papers are represented in the scatter plot, implying that the FS values range from 0 to 6 MPa and STS values range from 0 to 4 MPa. The normal Q-Q plot reveals that the incorporation of CNT via the fracture surface in the fractured zone has resulted in a noteworthy improvement in the mechanical properties of GPC. Furthermore, the homogenizer improved the ability of CNT particles to fill tiny and nano holes in the GP matrix and the nucleation effect of CNT particles, reinforcing the matrix and increasing the FS. CNT particles’ enhanced nucleation influence reinforces the matrix and therefore enhances FS. The filling and bridging actions of CNT particles are responsible for this improvement. Nanomaterial admixture can also enhance the FS of FA-GPC. They also demonstrated a 160% increase in the FS of an FA-GPC paste when 0.5 wt.% was used [81].

The influence of nanomaterials on crack-bridging is the most essential in enhancing the FS of FA-GPC. The normal Q-Q plot for tensile strength is shown in Figure 8a. For the analysis of the Q-Q plot, previous literature results data were used (Table 1). Tensile strength results show linear variation with sigma as 0.58865, representing only 58% of the linear variation data.

The normal Q-Q plot for flexural strength is shown in Figure 8b. For the Q-Q plot analysis, past literature results data were used (Table 1). Flexural strength results show linear variation with sigma as 0.89337, representing only 89% of the linear variation data. The addition of different nanomaterials demonstrated different characteristics and with variation in their mechanical performances, due to which there is variation in sigma values in both STS and FS.

### 3.4. Relationship between Bulk Density (BD) and Water Absorption (WA) of Nanomaterials

**Figure 9:**
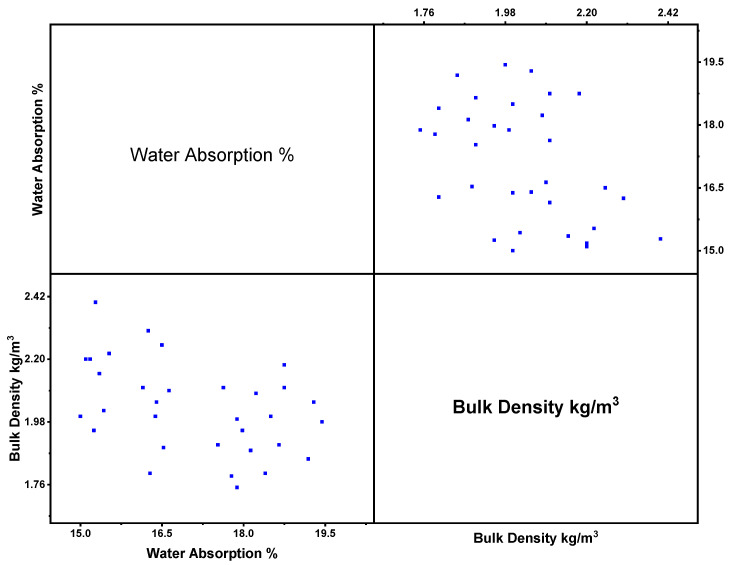
Scatter plot for WA (%) and BD (kg/m^3^).

Figure 9 shows the scatter plot for various nanomaterials for the properties of WA and BD. BD and WA values for GPC collected from various literature papers (Table 1) are represented in the Scatter plot, implying that the WA values range from 16% to 18.6% and BD values range from 1.78 to 2.42 g/cc. The well-homogenized dispersion of CNT in GPC improves particle packing, resulting in a much more compacted, denser microstructure that reduces water permeability via its matrix [15,82]. The addition of nanoparticles can reduce GP porosity. Because of the decreased porosity, the structure becomes more compact, and WA decreases. A water absorption by geopolymer is intimately dependent on its porosity and decrease as the amount of nanomaterials in the GP increases. The inclusion of nanoparticles can lower GP porosity. Decreased porosity results in a more compact structure and less water absorption by the geopolymer [22,32,83].

The morphological texture enhances with the concentration of nano owing to the filling of micro-voids. The addition of 2.5% NS resulted in a dense microstructure, possibly because of a faster hydration process and more nucleation sites accessible for GPC formation. On the other hand, a greater NS concentration promotes agglomeration, resulting in non-uniform silica particle dispersion, void formation, and loss of microstructure uniformity. When they introduced 2% NS to GBFS-mixed FA geopolymers, they noted the formation of a more compact and denser microstructure. The effect of NS on a metakaolin-based GPC with 5% wasted catalyst was recorded, with the formation of an unreacted particle bonding zone creating holes in the GP matrix. By accelerating the polymerization reaction and lowering nano porosity, 0.5% NS substantially enhanced matrix densification [24,84].

### 3.5. Microstructure Ansysis of Nanomaterial-Based GPC

#### 3.5.1. Scanning Electron Microscope (SEM)

**Figure 10:**
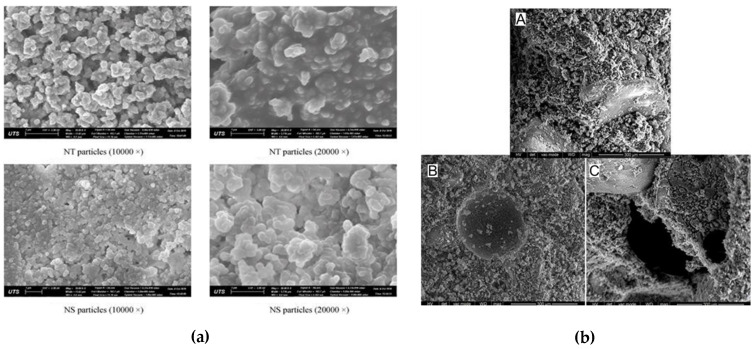
(**a**) SEM images of NT (nano titanium dioxide) and NS (nano silica) morphology of nanoparticles (reproduced Copyright 2022, with permission from Elsevier [74]); (**b**) SEM images of MVCNT with A: 0% MVCNT, B: 0.1% MVCNT, and C: 0.4% MVCNT. (Reproduced Copyright 2022, with permission from Elsevier [21].)

The microstructure of nanomaterial-based GP samples was investigated using SEM micrographs from prior studies are discussed here. Figure 10a,b represents the SEM images of NT, NS, and MVCNT in the GPC matrix. Even with a small quantity of NS added, the polymerization was aided, resulting in N–A–S–H gel forming, which improved strength [69,85]. When evenly dispersed in the solution, nanoparticles acted as a filler material, enhancing the hydration process and, as a result, improving the microstructure [86].

Furthermore, the silica fume (SF) has bigger particles and pozzolanic activity. Minimal-sized nanoparticles fill the pores in the GPC matrix and enhance its mechanical performance. It was observed that NS with a particle size of 40 nm could fill pores and disperse uniformly in the cement system. As a result, the microstructure becomes denser and more compressed one [27,87]. The CS result confirmed the conclusions of the microstructural investigation. The CS of NS with a particle size of 40 nm was higher than that of the particle sizes 12 and 20 nm. This is primarily due to agglomeration, caused by the poor dispersion of the smaller particles of 12 and 20 nm. Using NS to create a denser GP matrix microstructure, there are fewer unreacted FA particles in GPC. NS particles served as an inner filler, filling holes in the GPC matrix and helping to achieve the compaction [68,88].

With the addition of NS, the microstructure of an FA-based GPC was created in both wet and dry mixing conditions. The majority of the FA particles were transformed to Geopolymeric gel after 3.0 wt.% NS was added. In a nanocomposite GPC, there is a higher amount of amorphous material. The GP matrix made in dry mix conditions, on the other hand, contained fewer microcracks than the GPC matrix made in wet mix conditions. NS functioned better as a void filler in dry mixing settings than wet mixing [8,73,89].

NS has a very heterogeneous and porous microstructure, whereas slag-based GPC without NS has a highly heterogeneous and porous microstructure. Geopolymerization processes in GP products might benefit from carbon nanotubes (CNT). CNT enhances polymerization and densifies the microstructure of the GPC matrix. MVCNTs implement bridging micro-fractures in GP paste, showing that MVCNT and GP paste has exceptional bonding. Due to the consistent deployment of MVCNTs in the matrix, the GPC, with 0.1 wt.% MVCNT has a more compact and homogenous matrix than the 0 wt.% and 0.4 wt.%. At higher concentrations of MVCNTs, such as 0.4 wt.%, agglomerates form, resulting in the molecular chain [89].

#### 3.5.2. X-ray Diffraction (XRD)

**Figure 11:**
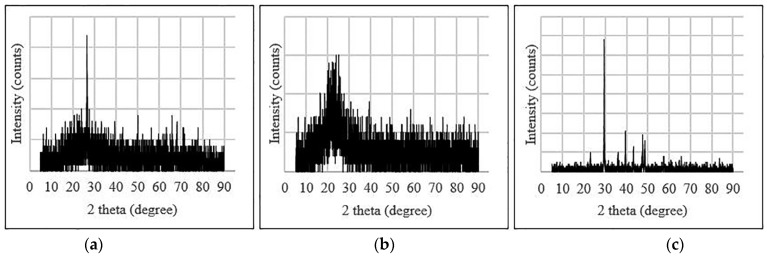
The XRD pattern for (**a**) fly ash (**b**) nano SiO_2_, and (**c**) nano-CaCO_3_ (reproduced Copyright 2022, with permission from Wiley [20]).

XRD analysis has been used in several studies to look into the impacts of NC and nano-CaCO_3_ inclusion on the chemical structure of GPC. Figure 11 shows the XRD pattern for SF, OPC, and nano-SiO_2_ and -CaCO_3_. Cloisite dominates the crystalline phase in NC, with traces of quartz and cristobalite were observed in XRD images. In addition to anatase, quartz, and mullite, albite was formed when nano-CaCO_3_ was added to the GP(M). Nano-CaCO_3_ in the GPC increases the crystalline phase intensity. This increased intensity is also a result of the addition of nano-CaCO_3_ to GPC, which has to speed up the geopolymerization rate [20,90].

Crystalline quartz was observed at 26–32 for 2 h due to the development of crystalline composition in GP matrices. The density of quartz in an FA-based GPC with nano silica has risen. GP compositions originated from nano silica, as shown by peaks at 2-theta in XRD graphs. GPC with an NS content of 6%. A few high peaks were found GPC when Nanomaterial was added, revealing the presence of SiO_2_, Ca_3_SiO_3_, and CaCO_3_ phases [91].

Nano silica into the GPC increases matrix density while improving mechanical properties [67]. The XRD pattern of GP paste with nano alumina admixed is equivalent to that of GP paste with NS added. According to the author, a similar XRD pattern may be seen in FA and GPC with or without nano alumina based on XRD analysis. This specifies that nano alumina does not play a substantial role in the same way NS does [92].

Nano alumina has minimal effect on polymerization in general. However, it may serve as a nano filler, enhancing the microstructure by filling pore spaces [93]. According to research, the inclusion of nano-TiO_2_ causes the development of more hydrated structures. Compared to the standard GP (without nano-CaCO_3_), an additional crystalline phase of albite (AB) was detected as peaks with a greater intensity implying a more crystalline structure in nano-CaCO_3_ added GPC [94].

#### 3.5.3. Fourier Transform Infrared Spectroscopy (FTIR)

**Figure 12:**
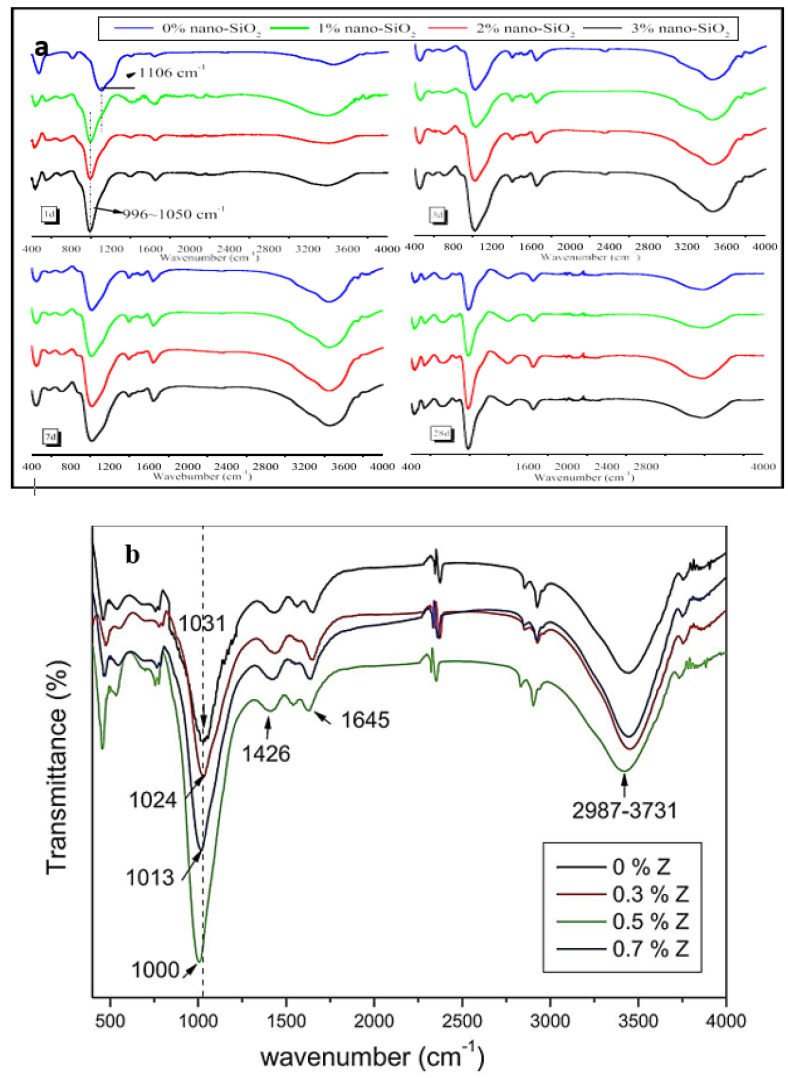
(**a**) The FTIR spectra for NS (reproduced Copyright 2022, with permission from Elsevier [33]); (**b**) shows the FTIR spectra for nano-ZnO (reproduced from [95]).

The FTIR spectra of GP with and without nanomaterials were reported in several prior studies to investigate the effect of NS and nano-ZnO (Figure 12). The effect of NS on the GPC FTIR indicated that NS caused a slight shift in the Si–O–Al vibration band to the lower transmittance in metakaolin-based GPC FTIR spectra. Figure 12a,b shows FTIR spectra for NS and nano-ZnO, respectively.

FTIR is frequently employed to examine the chemical components of typical cementitious materials hydration products and their relative amounts by changing the particular wavenumbers and transmittances. The absorption band in the FTIR spectra of blended GGPC did not change much, showing the impact of NS addition to the GGPC. There is no substantial variation in the location of terminal Si–O linkages in the reaction matrix owing to the preponderance of calcium from GGBS, limiting NS and FA effects on the GPC. As the amount of NC injected increases, the width of the main asymmetric band at wavenumbers about 1027–1032 cm^−1^ widens [3,95,96].

Due to the inclusion of Si and Al in polymerization, geopolymers with a small amount of NC showed bending and symmetric band removal. With a 7% increase in NC addition, the symmetric band of Si–O–Al is reduced. At wavenumbers of 1420, 946, and 457 cm^−1^, NT addition dramatically enhanced GPC transmittances, suggesting more carbonated products and polymerization products such as C–S–H and N–A–S–H. FTIR of GP exhibited a distinct intensity band between 1300 and 900 cm^−1^ when NS metakaolin-based GPC with different SiO_2_/Na_2_O ratios were included. FTIR analysis of a spent catalyst metakaolin-based GGPC with 0.5%, 1%, and 2% NS concentrations. The FTIR curves various pharmacological with metakaolin conversion levels ranging from 0% to 20%. In GP mixes containing 0% of NS, the peaks visible at 1300–900 cm^−1^ are attributable to Si–O–Al symmetrical vibrations [97].

## 4. Effect of Nanomaterials on Health Issues

Nanomaterials are frequently employed in the building industry due to their potential to improve the properties of cementitious materials. Various nanoscale sizes of these materials may pose a significant risk to human health if inhaled when dealing with them. Because nanoparticles may readily pass through the cell membrane without being endocytosed, they can disrupt cell growth in the human body by directly stimulating cell area, cell growth, and the cytoplasmic membrane. Some of the research conducted so far on the impacts of nanomaterial use suggests a severe concern about their impacts on the respiratory and cardiovascular systems, indicating a higher prevalence of asthma. NS with a diameter of 70 nm has been discovered to penetrate the skin and travel across the body via the lymphatic system, causing severe skin issues. CNC can potentially be harmful to the respiratory system. The degree of nanoparticle toxicity is undoubtedly affected by several factors, including the number of nanoparticles breathed in, the shape, particle size, surface area, and crystallinity [76,88,98].

NT and Al_2_O_3_ are more hazardous than their macro-sized counterparts among all nanoparticles. Nanoparticles are adequately managed during mixing or shipping, and health risks can be reduced while maximizing their use in the building industry. Furthermore, appropriate safety precautions must be taken while working with nanoparticles in the construction sector or a laboratory for characterization. Nanoparticles of NT and Al_2_O_3_ are considered more dangerous than their macro particle equivalents. However, if nanoparticles are adequately handled while mixing or shipping, health hazards can be avoided while their usage in the construction sector is maximized. Furthermore, while working with nanoparticles in the construction industry or laboratories for analysis, adequate safety procedures must be followed [6,64,99].

## 5. Practical Application of Nanomaterials

Usage of nanomaterials helps to reduce the usage of natural resources by increasing the performance of building materials and lowering energy consumption. Nanomaterials includes such as nano silica, nano alumina, nano clay, nano tube, multi-walled carbon nano tube, and nano-TiO_2_. These nanomaterials show excellent structural performance; they have the same applications as traditional concrete, such as construction of buildings, concrete road, paver, bricks, precast panels, concrete pipes, culverts, etc. The use of nanoparticles in construction provides a less expensive, quicker, and safer method of producing building materials. The expenses paid over the life cycle of nanomaterials can be decreased by increasing their technical qualities, and a more reasonable approach to the use of raw materials in construction can be accomplished. Product durability and efficiency may be improved, and raw material production performance levels can be raised. Nanotechnology has the capacity to turn the building industry into a period focused on environmental protection and innovative competitiveness, and it also has the potential to transform the construction industry into a period focused on sustainability [37,48,50,56,78,100].

Recently, the recycling of concrete containing nanoparticles was explored, and it was discovered to have a better compressive strength than recycled conventional concrete. The biggest difficulty with recycling concrete without nanomaterials is that it is inferior to conventionally made concrete in terms of durability and mechanical qualities. This is a problem when attempting to utilize recycled concrete for large-scale infrastructure and projects, which invites a higher risk and, as a result, may discourage the use of recycled concrete. However, it has been discovered that adding nanomaterials to recycled conventional concrete can generate mechanical qualities similar to normal concrete. The use of nanomaterials improves the strength and microstructure of concrete while decreasing its workability [59,101].

**Figure 13:**
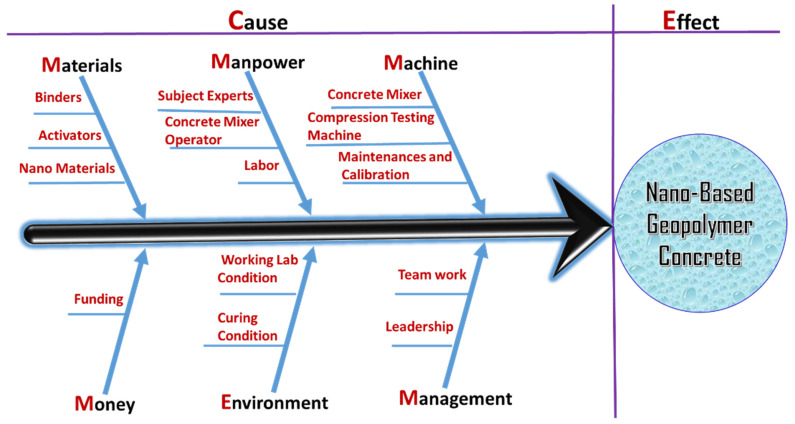
Ishikawa cause-and-effect diagram of GPC.

Major concerns have been expressed about the introduction of designed or inadvertent nanoparticles into the environment via a variety of mechanisms. It is critical to consider nanomaterial pathways from industry to the environment and to do all that is possible to reduce emissions. There is no structure or necessity for the sector to accomplish nanomaterials release targets due to a lack of legislation controlling the particular release of nanomaterials into the environment. A better knowledge of the functions that nanoparticles play in the environment, as well as the harmful impacts of exposure to these particles in various contexts, is required. As a result, a thorough evaluation of the current literature, as well as more rigorous study into the biological and environmental interactions with nanoparticles, is essential [102]. Figure 13 shows the Ishikawa cause-and-effect diagram of GPC.

## 6. Conclusions

Nanomaterials shows excellent structure performance when used as binders, with small dosage of nanomaterials such as nano silica, nano alumina, nano clay, nano tubes, multi-walled carbon nano tubes, accelerating the rate of geopolymerization process.

1. The inclusion of nanoparticles into geopolymers considerably lowers the geopolymers setting time. Nanomaterials are particularly useful in making up for the limitations of ambient-cured GPC.
2. Incorporating nanomaterials such as nano silica, nano alumina, and nano clay into the GPC composite morphology significantly refines the structural stability of the GPC composite, improving its durability. The various products in GPC composites emerging from the incorporation of highly reactive nanoparticles also contribute significantly to geopolymer durability by sealing the holes and cracks in its matrix.
3. The microstructure of geopolymer-containing nanoparticles was more compact and uniform. Nanomaterials in geopolymers enhance the polymerization rate, according to SEM, XRD, and FTIR analysis. Moreover, these characterization tests show that adding NS, CNT, NT, and NCC to GP does not form new phases.
4. Nanoscale materials provide new possibilities in a wide range of sectors, with applications that are diverse and expanding all the time. As a result, numerous sectors have embraced the benefits that nanoparticles may give, propelling industry-specific products forward. This may be seen in a wide range of applications, including building with nanoparticle-enriched recycled materials that have similar mechanical qualities to new materials, medicine delivery, and antibacterial capabilities in clothes.
5. The nanomaterials demonstrated remarkable reactivity; they can be used as superfine pozzolanic materials in GPC composites to improve the mechanical properties of geopolymers by attaining a higher degree of hydration. High nanomaterial dosage in GPC results in a lower polymerization process level due to insufficient dissolving and possible material agglomerate, resulting in lower structural performances of GPC.

## Figures and Tables

**Table 1:** Shows the use of nanomaterials as additives in binders.

Binder	Size	% of Variation	Max. CS/STS/FS/WA	Tests	Molarity	Code	References
Kaoline + Silica fume	50 mm^3^	5, 10, 15	10%–46 MPa	CS, EDS, XRD	-	ASTM C109	[45]
Metakaolin + NS	40 mm^3^	1, 2, 3	2%–28 MPa	Efflorescence, CS, WA, FTIR, SEM, EDX, XRD	-	ASTM C109	[4]
FA + NS + Micro Silica	-	5, 10, 15	5%–15.2 MPa	Flow test, CS, WA, FTIR, XRD	-	ASTM C109, Flow test-ASTM1437-16WA-ASTM, C1403-15	[46]
FA + Pumice (Sand)	50 mm^3^-CS, 40 × 40 × 160 mm-FS	35	14 M–29 MPa-CS 14 M–4 MPa-FS	Flow tests, CS, FS, Ultrasonic pulse velocity, Rheology	10, 12, 14	CS-ASTM C109 Flow Test-ASTM C 1437, FS-ASTM C348-18 UPV-ASTM C597-16	[42]
FA + NS + NCC	40 × 40 × 160, mm-FS	1, 2, 3	12 M–69.7 MPa-CS 12 M–10.7 MPa-FS	CS, FS, FESEM, Ultrasonic pulse velocities	8, 10, 12	FS-TS EN 1015–11, Flow test-TSEN 1015–3	[8]
Metakaolin + NZ	Dia. of 30 mm and ht. of 60 mm-CS	0.3, 0.5, 0.7	0.5%–38 MPa	CS, SEM, XRD, FTIR, RXF, MIP, thermogravimetric, Density and WA	10	WA-ASTM C140, CS-ASTM D1633-00	[47]
FA + GGBS + NS	70.6 mm^3^-CS,	0, 1, 2	2%–70.20 MPa-CS 2%–5.22 MPa-STS, 2%–5.57 MPa-FS	CS, STS, FS, WA, RCPT, Water Sorptivity	3	CS-ASTM (2001) C 109, STS-ASTM (2001) C496, FS-ASTM (2001) C 78	[48]
FA + GGBS + Glass bottles waste Nano powder	50 mm^3^ CS, 40 × 40 × 160, mm-FS, 150(L), 75(D) mm-STS	5, 10, 15, 20	5%–65 MPa-CS, 5%–6.8 MPa-FS, 5%–4.7 MPa-STS, 20%–10.2%-WA	Flow test, CS, STS, FS, TGA, FTIR, SEM, XRD, WA	2	FS-ASTM C78, STS-ASTM C496/C496M-11, CS-ASTM C109/109M	[41]
FA + NS + NT	50 mm^3^-CS	2%	35.8 MPa and 33.7 MPa for NS + NT	CS, XRD, SEM, TGA, Hydration heat test	-	CS-ASTM C109	[49]
FA + GGBS + NS	75(D) × 150 (H) mm-CS	0, 0.5, 1, 1.5, 2.0, 2.5, 3.0	2.5%–53.2 MPa-CS	Flow test, CS, ATR-FTIR, FESEM, TGA	10	Flow tests-ASTM C1437	[26]
FA + OGPC + GGBS + NS	50 mm^3^-CS	0, 0.5, 1, 1.5, 2.0, 2.5, 3.0	3%–56 MPa	Flow, CS, SEM, EDX, XRD	8	CS-ASTM C109, C1437	[27]
FA + NT	40 mm^3^-CS	1, 3, 5	5%–22% higher than reference sample	Workability, CS, SEM, XRD	10	Workability-ASTM C230	[50]
Metakaolin + NS	-	0, 1, 2, 3	1%–15.8 MPa-FS	FS, MAS NMR, TG/DTA	-	-	[28]
Metakaolin + NS	70.6 mm^3^-CS	0, 2, 4, 6, 8, 10	4%–52.77 MPa-CS	CS, SEM	14	-	[31]
Air cooled slag + Water cooled slag + MVCNT	25 mm^3^-CS	0.1, 0.2, 0.3	0.1%–20.5 MPa-CS	SEM, XRD, FTIR	6	-	[7]

## Data Availability

The data that support the findings of this study are available from the corresponding author, upon reasonable request.
